# Supplementary material for: HIVprotI: an integrated web based platform for prediction and design of HIV proteins inhibitors
Source: J Cheminform. 2018 Mar 9;10:12. doi: 10.1186/s13321-018-0266-y (PMC5845081; doi:10.1186/s13321-018-0266-y)
Supplement: Supplementary file 2 — Additional file 2. Source code of HIVProtI web server. [file 13321_2018_266_MOESM2_ESM.zip › HIVprotI_Source-code/hivprotI/weka-3-6-6/doc/weka/associations/gsp/Sequence.html]

Sequence


---


|  |  |  |
| --- | --- | --- |
| **Overview** \| **Package** \| **Class** \| **Tree** \| **Deprecated** \| **Index** \| **Help** \| **Weka's home** | |  |
| **PREV CLASS**   NEXT CLASS | **FRAMES**    **NO FRAMES**     **All Classes** |  |
| SUMMARY: NESTED \| FIELD \| CONSTR \| METHOD | DETAIL: FIELD \| CONSTR \| METHOD |  |


---


## weka.associations.gsp Class Sequence

```
java.lang.Object
  weka.associations.gsp.Sequence
```

**All Implemented Interfaces:**: java.io.Serializable, java.lang.Cloneable, RevisionHandler

---

``` public class Sequence extends java.lang.Object implements java.lang.Cloneable, java.io.Serializable, RevisionHandler ```

Class representing a sequence of elements/itemsets.

**Version:**
:   $Revision: 1.2 $

**Author:**
:   Sebastian Beer

**See Also:**: Serialized Form

---

**Constructor Summary**

`Sequence()`
  
          Constructor.

`Sequence(FastVector elements)`
  
          Constructor accepting a set of elements as parameter.

`Sequence(int supportCount)`
  
          Constructor accepting an int value as parameter to set the support count.


| **Method Summary** | |
| --- | --- |
| `static FastVector` | `aprioriGen(FastVector kMinusOneSequences)`             Generates all possible candidate k-Sequences and prunes the ones that contain an infrequent (k-1)-Sequence. |
| `Sequence` | `clone()`             Returns a deep clone of a Sequence. |
| `static FastVector` | `deleteInfrequentSequences(FastVector sequences, long minSupportCount)`             Deletes Sequences of a given set which don't meet the minimum support count threshold. |
| `boolean` | `equals(java.lang.Object obj)`             Checks if two Sequences are equal. |
| `java.lang.String` | `getRevision()`             Returns the revision string. |
| `static FastVector` | `oneElementsToSequences(FastVector elements)`             Converts a set of 1-Elements into a set of 1-Sequences. |
| `static void` | `printSetOfSequences(FastVector setOfSequences)`             Prints a set of Sequences as String output. |
| `static java.lang.String` | `setOfSequencesToString(FastVector setOfSequences, Instances dataSet, FastVector filterAttributes)`             Returns a String representation of a set of Sequences where the numeric value of each event/item is represented by its respective nominal value. |
| `java.lang.String` | `toNominalString(Instances dataSet)`             Returns a String representation of a Sequences where the numeric value of each event/item is represented by its respective nominal value. |
| `java.lang.String` | `toString()`             Returns a String representation of a Sequence. |
| `static void` | `updateSupportCount(FastVector candidates, FastVector dataSequences)`             Updates the support count of a set of Sequence candidates according to a given set of data sequences. |

**Methods inherited from class java.lang.Object**

`getClass, hashCode, notify, notifyAll, wait, wait, wait`

**Constructor Detail**

### Sequence

```
public Sequence()
```

:   Constructor.

---


### Sequence

```
public Sequence(FastVector elements)
```

:   Constructor accepting a set of elements as parameter.

    **Parameters:**: `elements` - the Elements of the Sequence

---


### Sequence

```
public Sequence(int supportCount)
```

:   Constructor accepting an int value as parameter to set the support count.

    **Parameters:**: `supportCount` - the support count to set


**Method Detail**

### aprioriGen

```
public static FastVector aprioriGen(FastVector kMinusOneSequences)
                             throws java.lang.CloneNotSupportedException
```

:   Generates all possible candidate k-Sequences and prunes the ones that
    contain an infrequent (k-1)-Sequence.

    :   **Parameters:**: `kMinusOneSequences` - the set of (k-1)-Sequences, used for verification **Returns:**: the generated set of k-candidates **Throws:**: `java.lang.CloneNotSupportedException`

---


### deleteInfrequentSequences

```
public static FastVector deleteInfrequentSequences(FastVector sequences,
                                                   long minSupportCount)
```

:   Deletes Sequences of a given set which don't meet the minimum support
    count threshold.

    :   **Parameters:**: `sequences` - the set Sequences to be checked: `minSupportCount` - the minimum support count **Returns:**: the set of Sequences after deleting

---


### oneElementsToSequences

```
public static FastVector oneElementsToSequences(FastVector elements)
```

:   Converts a set of 1-Elements into a set of 1-Sequences.

    :   **Parameters:**: `elements` - the set of 1-Elements **Returns:**: the set of 1-Sequences

---


### printSetOfSequences

```
public static void printSetOfSequences(FastVector setOfSequences)
```

:   Prints a set of Sequences as String output.

    :   **Parameters:**: `setOfSequences` - the set of sequences

---


### setOfSequencesToString

```
public static java.lang.String setOfSequencesToString(FastVector setOfSequences,
                                                      Instances dataSet,
                                                      FastVector filterAttributes)
```

:   Returns a String representation of a set of Sequences where the numeric
    value of each event/item is represented by its respective nominal value.

    :   **Parameters:**: `setOfSequences` - the set of Sequences: `dataSet` - the corresponding data set containing the header information: `filterAttributes` - the attributes to filter out **Returns:**: the String representation

---


### updateSupportCount

```
public static void updateSupportCount(FastVector candidates,
                                      FastVector dataSequences)
```

:   Updates the support count of a set of Sequence candidates according to a
    given set of data sequences.

    :   **Parameters:**: `candidates` - the set of candidates: `dataSequences` - the set of data sequences

---


### clone

```
public Sequence clone()
```

:   Returns a deep clone of a Sequence.

    :   **Overrides:**: `clone` in class `java.lang.Object`
    :   **Returns:**: the cloned Sequence

---


### equals

```
public boolean equals(java.lang.Object obj)
```

:   Checks if two Sequences are equal.

    :   **Overrides:**: `equals` in class `java.lang.Object`
    :   **Returns:**: true, if the two Sequences are equal, else false

---


### toNominalString

```
public java.lang.String toNominalString(Instances dataSet)
```

:   Returns a String representation of a Sequences where the numeric value
    of each event/item is represented by its respective nominal value.

    :   **Parameters:**: `dataSet` - the corresponding data set containing the header information **Returns:**: the String representation

---


### toString

```
public java.lang.String toString()
```

:   Returns a String representation of a Sequence.

    :   **Overrides:**: `toString` in class `java.lang.Object`
    :   **Returns:**: the String representation

---


### getRevision

```
public java.lang.String getRevision()
```

:   Returns the revision string.

    :   **Specified by:**: `getRevision` in interface `RevisionHandler`
    :   **Returns:**: the revision


---


|  |  |  |
| --- | --- | --- |
| **Overview** \| **Package** \| **Class** \| **Tree** \| **Deprecated** \| **Index** \| **Help** \| **Weka's home** | |  |
| **PREV CLASS**   NEXT CLASS | **FRAMES**    **NO FRAMES**     **All Classes** |  |
| SUMMARY: NESTED \| FIELD \| CONSTR \| METHOD | DETAIL: FIELD \| CONSTR \| METHOD |  |


---
